# Taste Responses in the Nucleus of the Solitary Tract of Awake Obese Rats Are Blunted Compared With Those in Lean Rats

**DOI:** 10.3389/fnint.2019.00035

**Published:** 2019-07-30

**Authors:** Michael S. Weiss, Andras Hajnal, Krzysztof Czaja, Patricia M. Di Lorenzo

**Affiliations:** ^1^Department of Psychology, Binghamton University, Binghamton, NY, United States; ^2^Department of Neural and Behavioral Sciences, College of Medicine, The Pennsylvania State University, Hershey, PA, United States; ^3^Department of Veterinary Biosciences and Diagnostic Imaging, University of Georgia, Athens, GA, United States

**Keywords:** high fat diet, obesity, taste, nucleus of the solitary tract, electrophysiology, gustatory, rat

## Abstract

Taste perception changes with obesity but the underlying neural changes remain poorly understood. To address this issue, we recorded taste responses from single cells in the nucleus tractus solitarius (NTS, the first synapse in the central gustatory circuit) in awake, diet-induced obese [(DIO; ≥ 8 weeks on a high-energy diet (45%fat, 17% sugar; HED)], and lean rats. Rats were implanted with a bundle of microelectrodes in the NTS and allowed to recover. Water-deprived rats were allowed to freely lick various tastants in an experimental chamber. Taste stimuli included an array of sapid stimuli dissolved in artificial saliva (AS). Each taste trial consisted of five consecutive licks followed by five AS licks presented on a VR5 schedule. Results showed that taste responses (*n* = 49 for DIO; *n* = 74 for lean rats) in NTS cells in DIO rats were smaller in magnitude, shorter in duration, and longer in latency that those in lean rats. However, there were proportionately more taste-responsive cells in DIO than in lean rats. Lick coherence in DIO rats was significantly lower than in lean rats, both in taste-responsive, and lick-related cells (*n* = 172 in lean; *n* = 65 in DIO). Analyses of temporal coding showed that taste cells in DIO rats conveyed less information about taste quality than cells in lean rats. Collectively, results suggest that a HED produces blunted, but more prevalent, responses to taste in the NTS, and a weakened association of taste responses with ingestive behavior. These neural adaptations may represent both negative effects and compensatory mechanisms of a HED that may underlie deficits in taste-related behavior associated with obesity.

## Introduction

The idea that obesity, an epidemic for United States adults (Ogden et al., 2015), is caused by a chronic surplus of energy from food belies the complexity of its etiology, and the obesity-related changes that occur in the body. Along with a constellation of hormonal and physiological changes that accompany obesity, alterations in the taste system are also evident. For example, an increase in visceral fat is negatively correlated with both olfactory and taste function (Fernandez-Garcia et al., 2017). In both humans and rodents, taste sensitivity changes with body weight, specifically for taste qualities that signal high-energy availability, e.g., sweet and fatty (Hajnal et al., 2005; Bartoshuk et al., 2006; Shin et al., 2011; Zhang et al., 2011, 2013; Duca et al., 2014; Dando, 2015).

The gustatory system serves as a feed-forward regulator of the ingestive neural circuitry. Taste serves as a sensory gate-keeper, eliciting different sensations depending on a food’s chemical composition (Scott and Mark, 1987; Spector and Travers, 2005; Chandrashekar et al., 2006), and acting as a mediator of food consumption (Berthoud, 2002; Zocchi et al., 2017). Thus, it is unsurprising that there are multiple taste-related neural structures that are sensitive to an animal’s internal state. For example, a high protein diet (Darcel et al., 2005), changes in blood glucose (Giza and Scott, 1983), changes in blood insulin (Giza and Scott, 1987), gastric distention (Baird et al., 2001), and obesity (Kovacs and Hajnal, 2008) can all alter taste processing in the brain.

The nucleus tractus solitarius (NTS), the first synapse in the mammalian central gustatory pathway, is a point of convergence for taste processing, and signals that inform the organism of its internal state. NTS neurons express a wide array of receptors involved in the homeostatic regulation of ingestion (Ritter et al., 2000; Faulconbridge et al., 2003; Rozengurt, 2006; Cui et al., 2011; Blouet and Schwartz, 2012; Zhan et al., 2013). Moreover, there is a large degree of intranuclear connectivity bridging the caudal viscerosensory pole with the rostral orosensory pole (Beckman and Whitehead, 1991; Ganchrow et al., 2014; Travers et al., 2018). With reciprocal connectivity from forebrain structures controlling feeding behavior such as the amygdala and hypothalamus (Whitehead et al., 2000) along with parallels in the vagal-visceral and lingual pathways (Karimnamazi et al., 2002), the gustatory system is a highly interactive and integrative system, with the NTS being a crucial first node in the feeding circuit.

Here, we aimed to detail the effects of obesity on responses to taste stimuli in the NTS. We recorded taste responses from single cells in the NTS of awake, freely licking rats that had been made obese by exposure to a high energy diet (HED), i.e., diet-induced obesity (DIO). We then compared cellular responses to taste and licking in DIO rats to those in lean rats maintained on standard lab chow. Results showed that taste responses in DIO rats are blunted compared with those in lean rats and their taste-evoked spike trains convey less information about taste quality. In addition, the relative proportion of taste cells was increased in the NTS of DIO rats, suggesting a compensatory mechanism for diminished taste responsivity in DIO rats.

## Materials and Methods

### Subjects

Male Sprague-Dawley rats (*N* = 78) were obtained from Taconic Labs, Inc. (Germantown, NY, United States) and allowed to acclimate to the vivarium for a minimum of 7 days with a 12 h light/dark cycle (lights off at 0900 h). Animals were then randomly divided into two groups, where one group (diet-induced obese, DIO; *n* = 39) was fed a high-fat, high sugar diet (HED; 45% kCal from fat, 17% sucrose, Research Diets D12451, New Brunswick, NJ, United States) starting at 8 weeks of age for a minimum of 8 weeks before surgical implantation and throughout testing until they were sacrificed. The lean control group (*n* = 39) was fed a standard chow diet (Purina Lab Diet 5001, 13.5% kCal from fat, 3.7% sucrose, Lab Diet, St. Louis, MO, United States) for the duration of the experiment. Although all rats in both the DIO and lean groups were surgically implanted with microelectrode bundles, we only obtained well-isolated cells from 14 lean and 7 DIO rats. For the remaining rats that were implanted with microelectrode arrays, the electrodes either missed their target or the recordings did not yield good quality recordings, e.g., some had movement artifact, others simply did not show any channels with well-isolated cells.

All procedures were in accord with the National Institutes of Health Animal Welfare Guide and were approved by the Institutional Animal Care and Use Committee of Binghamton University.

### Body Composition Monitoring

Before being placed on a HED, and after 8 weeks on a HED, DIO animals underwent a dual-energy X-ray absorptiometry (DXA) scan to determine body composition. Lean rats were DXA scanned within a day of the DIO rats at both time points. Animals were sedated with Medetomidine HCl (Pfizer Inc., NY, United States; 0.1 mg/kg, s.c.) and placed on a scanning bed where body composition was calculated by Hologic APEX Discovery A software (Hologic, Bedford, MA, United States). Scan results determined both body fat and lean tissue mass. Sedation was reversed by Atipamezole (Pfizer Inc., NY, United States; 0.1 mg/kg, i.p.) upon completion of the scan. At the beginning of the experiment, there were no significant differences in body weight [*t*(19) = 1.461, *p* = 0.160)] or percent body fat [*t*(19) = 0.783, *p* = 0.443] between those rats that supplied data for the Lean and DIO groups.

### Taste Stimuli and Delivery System

Taste stimuli included both traditional and “naturalistic” stimuli. Because we have shown that cells in the NTS of awake rats respond to both taste and odor stimuli (Escanilla et al., 2015), we hypothesized that the optimal stimuli for NTS cells would be those that represented actual food, i.e., naturalistic tastants. In fact, taste-responsive cells in the parabrachial nucleus of the pons, a structure to which the NTS sends its main projection, convey more information about naturalistic taste stimuli compared with traditional tastants (Sammons et al., 2016). We therefore decided to test both traditional and naturalistic tastants in the present study. Traditional taste stimuli in the present study included: 0.05M NaCl (N), 0.1 M and 0.5 M sucrose (Lo Su and Hi Su, respectively), 0.01 M citric acid (CA), and 0.002 M caffeine (Caf). These solutions were made from reagent grade chemicals purchased from Sigma-Aldrich (St. Louis, MO, United States) or Thermo Fisher Scientific (Pittsburg, PA, United States). Naturalistic taste stimuli included clam juice (Clm J; containing 0.12 M NaCl), 25 and 100% grape juice (Lo GJ and Hi GJ, respectively; containing 0.12 and 0.48 M sucrose), lemon juice (Lm J; containing 0.01 M citric acid), coffee (Coff; containing 0.002 M caffeine), and 25% heavy cream (Crm; to serve as a fat taste stimulus). All stimuli were dissolved in artificial saliva (AS; 0.015 M NaCl, 0.022 M KCl, 0.003 M CaCl2; 0.0006 M MgCl2; pH ∼ 7.4; Hirata et al., 2005; Breza et al., 2010).

Electrophysiological testing was conducted in an operant chamber (MED Associates, St. Albans, VT, United States), where rats could navigate freely. Pressurized (∼11 psi) 35 mL tubes mounted on the exterior of the testing chamber served as reservoirs for tastant solutions. Tastant reservoirs were connected to 20-gauge stainless steel tubes housed within a larger stainless steel tube (8 mm dia.) to form a single lick spout. Delivery of tastant solutions was controlled by solenoids (Parker-Hannifin, Fairfield, NY, United States) triggered by licks. Stimulus trials were controlled by MED Associates software (MED Associates, St. Albans, VT, United States). Lick-evoked fluid delivery was individually calibrated daily for each tastant reservoir so that 12 ± 1 μl of fluid were delivered immediately after the animals’ tongue broke an infrared beam located in front of the lick spout.

Stimulus delivery for electrophysiological experiments was as follows: a stimulus trial consisted of a block of five consecutive taste stimulus licks, separated by five “rinse” (AS) licks, each of which was presented on a variable ratio 5 schedule. That is, each AS lick was separated by four to six “dry” (non-reinforced) licks, except when AS serves as a “taste” stimulus. Taste stimulus trials were presented in a pseudorandomized order; to encourage the animal to lick the tastant delivery spout, Lo Su (0.1M sucrose) was the first stimulus encountered by the animal in each session.

### Microwire Implantation Surgery

Animals underwent a surgical procedure to implant microelectrode recording electrodes, performed under aseptic conditions. Animals were given 0.05 mg/kg Buprenorphine HCl and 0.05 mg/kg Atropine Sulfate (VETone, Boise, ID, United States) subcutaneously approximately 1 h before being anesthetized. Anesthesia was induced with 4% isoflurane and the dosage was tapered down throughout the surgery (1–3% maintenance). Vital signs (e.g., blood oxygenation, heart rate, respiration rate, and body temperature) as well as reflexive behaviors (e.g., toe pinch reflex) were monitored throughout the surgery to ensure a surgical plane of anesthesia. Body temperature was maintained at 37^∘^C by an autoregulating heating pad connected to a rectal thermistor. Artificial tear gel (AltaLube, Altaire Pharmaceuticals, Aquebogue, NY, United States) was applied to the eyes to prevent drying. The crown of the head was shaved and the animal was then placed in the stereotaxic apparatus (Kopf Model 1900, Tujunga, CA, United States).

Once the animal was in the stereotaxic instrument, the head was swabbed with Betadine / iodine scrub followed by a 70% ethanol solution three times and the animal was given an injection of 5 mL sterile lactated Ringers saline solution s.c. (Hospira, Lake Forest, IL, United States). An incision was then made along the midline and the underlying fascia was gently retracted. Six pilot holes were then drilled for implantation of bone screws, which were used as anchors for the dental acrylic head-cap. Additionally, one skull screw served as an electrical ground for the microwire electrode array. The head was then angled so that bregma was 25^∘^ below lambda. A small hole (∼2 mm dia.) was drilled in the occipital bone (∼14–15.3 mm posterior to bregma and ∼1.5–2.5 mm lateral to the midline) and the underlying dura was punctured and resected for the insertion of the microwire electrode assembly. The microwire assembly consisted of a bundle of 16 polyimide-insulated tungsten (25 μm dia., equal length) microwires attached to a drivable device enabling 4 mm extension (Innovative Neurophysiology, Durham, NC, United States). For implantation of the chronic microdrive, the probe was implanted at ∼4.5 mm ventral to the cerebellar surface, and the shuttle mechanism on the probe was used to advance the wires to the target post-operatively. The hole was sealed with a biocompatible silicone elastomer (Kwik-Cast, WPI Instruments, Sarasota, FL, United States) and the electrode was secured with an acrylic resin. Once the acrylic cured and the electrode was secured to the head, the animal received a 10 mL injection of warmed saline and was given 100% oxygen @ 1.5 L/min until the animal was spontaneously mobile. Once the animal was ambulatory, Buprenorphine HCl (0.05 mg, s.c.) and Gentamicin (6 mg/kg, s.c.) were given as an analgesic and antibiotic, respectively, for 3 days. Topical antibiotic with pain reliever (Neosporin) was applied around the head-cap daily for 5 days to prevent infection. Animals in post-operative care were given sterile isotonic saline or a lactated ringer’s solution (10–15 mL s.c.) as needed to prevent dehydration. Animals were kept in post-operative recovery for a minimum of 7 days and until they regained their pre-operative weight.

In addition to the procedures outlined above, several accommodations were added for DIO rats undergoing electrode implantation. First, additional time was allocated between the initial anesthesia induction and head mounting in the stereotaxic instrument. Second, the animal was placed on the heating pad for 15–20 min prior to surgery. Third, following surgery, DIO rats were given extra lactated ringers solution (10–15 ml at a time, s.c.) and Buprenorphine HCl (0.1–0.15 ml, s.c.).

### Electrophysiological Recordings

Prior to testing, animals were water deprived for 22 h. Animals were placed in an operant chamber and a head-stage and cable were attached to the animals’ electrode assembly. A house light inside the operant chamber signaled the beginning of a recording session. Neural activity (timestamps of single waveforms, with 25 μs resolution) as well as the identity of stimulus deliveries were obtained using Sort Client software (Plexon, Dallas, TX, United States). After recording sessions, waveforms were exported to Offline Sorter (Plexon, Dallas, TX, United States) for individual unit isolation. Criterion for isolation was a refractory period of > 2 ms and distinct clusters in principal component feature space (Stapleton et al., 2006). When more than one unit was recorded on the same day, albeit from different wires, we applied a cross-correlation function (CCF) to all possible pairs of units. Those units that showed a narrow peak at exactly zero in the CCF were classified as the same unit and only one recording was included in subsequent analyses. After recording sessions, animals were returned to their home cages. After 1 h in their home cages, rats were given 1 h of water access. Each animal experienced repeated daily (except for weekends) recording sessions until there were no more isolatable units and/or until the electrode assembly was past the NTS; animals were run for several weeks.

### Data Analysis – General

Spontaneous firing rate was determined by obtaining the mean and standard deviation (SD) of firing rate (in spikes/sec; sps) from multiple 14 s samples during which there was no licking. The first 3 s of the 14 s-window served to ensure that there was no stimulus left in the mouth to exclude any potential stimulus-evoked firing. The next 10 s is the period where spontaneous firing rates were calculated. The 1 s-window after the spontaneous period was calculated served to ensure there was no firing rate change due to anticipatory behavior of licking. Baseline firing rate for a given taste stimulus was calculated as the average firing rate (in sps) during the 1 s of activity before the first stimulus lick across all trials.

Responses were calculated for two time scales: 5-lick responses where the first lick (of five stimulus licks served as time zero and the response interval extended over 4 s, and lick-by-lick responses where each taste stimulus lick served as time zero and the response interval extended over 170 ms.

For 5-lick responses, taste stimulus-evoked activity was defined as a significant change in firing rate from baseline activity. A significant change from baseline was determined by taking a 100 ms window, moved in 20 ms increments until the firing rate was 2 SDs (95% confidence interval) above, or below the baseline firing rate for three consecutive 100 ms bins. The trialing edge of the first bin that was significantly above baseline was defined as the response latency. Once this bin was identified, the 100 ms window was moved until the response was no longer significantly different from baseline. Response magnitude was determined by subtracting the taste-evoked firing rate from baseline firing rate.

For lick-by-lick responses, a significant taste response was calculated by taking a 10 ms window, moved in 2 ms increments until the firing rate was 2 SDs above or below the baseline firing rate for three consecutive 10 ms bins. The baseline firing rate was calculated from a 50 ms pre-trial window. The trialing edge of the first bin that was significantly above baseline was defined as the response latency. Once this bin was identified, the 10 ms window was moved until the response was no longer significantly different from baseline. Response magnitude was determined by subtracting the taste-evoked firing rate from baseline firing rate.

Responses to taste stimuli were defined as excitatory if the firing rate during the taste stimulus significantly exceeded the firing rate of the cell in the baseline period immediately preceding the initiation of the taste stimulus. Conversely, inhibitory responses were defined by significant decreases in firing rate with respect to baseline firing rate.

To determine if differences existed between the proportional frequency of neurons responding on a lick-by-lick basis, 5-lick basis, or both, a Mann-Whitney *U* Test was applied.

The degree to which firing patterns of NTS cells tracked the lick pattern, a measure of lick coherence was calculated using Neuronexus software (Plexon, Dallas, TX, United States). The maximum coherence value in the 4–9 Hz range was taken as the lick coherence value for a given cell. Lick coherence values ranged from 0 (spike activity and licks were independent) to 1 (spiking was time-locked to licks). Differences in the distribution of lick coherence were compared using a Mann-Whitney *U*-Test.

Other statistical tests used are described throughout the section “Results.” Statistical calculations were done using GraphPad Prism 7.0 software. For all statistical tests, an α = 0.05 was considered significant.

### Data Analysis – Temporal Coding

To assess the contribution of the temporal characteristics of a taste-evoked spike train to taste coding, metric space analysis (MSA; Victor and Purpura, 1996; Purpura and Victor, 1997) was used. This analytic approach was used previously (Roussin et al., 2012; Weiss et al., 2014; Escanilla et al., 2015; Sammons et al., 2016) and is described briefly here.

MSA first quantifies the dissimilarity, or distance, between spike trains calculated as the “cost” of transforming one spike train into another by adding, removing, or moving spikes in time. The first metric is called *D^*count*^*, where moving spikes in time has no cost; adding or deleting spikes has a cost of 1. This measure is simply the difference in the number of spikes between two taste-evoked spike trains. A second metric, *D*^*spike*^[*q*], takes the timing of individual spikes into account. Here, the cost of adding of deleting spikes is parameterized by *q*, a measure of temporal precision, such that the cost of moving a spike in time is *qt; q* is in units of 1/q s.

To estimate the amount of information (*H*) conveyed by spike trains, the degree to which pairs of responses to the same stimulus differed from pairs of responses to different stimuli was determined at varying levels of temporal precision (*q*) by calculating the mutual information between the inferred stimulus labels, and the true stimulus labels. The level of *q* at which information is greatest is termed *H*_*max*_. Information when *q* is set to 0 is termed *H*_count_, indicating the amount of information contributed by spike count alone.

Two analyses constructed from synthetic data sets served as controls. Shuffled analyses randomly assigned stimulus labels to taste responses and recalculated information. These analyses were performed 40 times at each level of *q*. The resulting information value was termed *H*_*shuffled*_. Exchange analyses assessed the extent to which the rate envelope of the response is informative; spikes were reordered randomly in time while preserving the progression in time of the overall firing rate in the response. This process was repeated 10 times for each level of *q*. Information resulting from this analysis was termed *H*_exchange_. If *H*_*max*_ was greater than *H*_shuffled_ +2SD, it was concluded that the taste-evoked spike trains conveyed a significant amount of information about taste quality. The circumstance where *H*_*max*_ was greater than *H*_shuffled_+2SD and equal to *H*_count_ implied that the taste-evoked firing rate was more informative that either the rate envelope or spike timing. If *H*_*max*_ was greater than *H*_shuffled_ +2SD but not greater than *H*_exchange_, it was concluded that the rate envelope of a response conveyed a significant amount of information about taste quality. Finally, if *H*_*max*_ exceeded *H*_shuffled_ +2SD and *H*_exchange_, it was concluded that spike timing contributes to the neuron’s ability to distinguish among taste stimuli.

To assess the contribution of temporal and rate coding in NTS cells from DIO and lean rats, the average amount of information across the entire sample was calculated at 200, 500, 1000, 1500, and 2000 ms of response. First, the amount of information conveyed about taste quality was calculated using responses to 0.1 M sucrose, 0.05 M NaCl, 0.01 M citric acid, and 0.002 M caffeine for traditional taste stimuli and 25% grape juice, 75% clam juice, lemon juice, and coffee for naturalistic taste stimuli. At each time point, the amount of information (*H*_*max*_, in bits) conveyed by each cell where *H*_*max*_ > *H*_*shuffled*_+2SD was calculated. For cells that did not meet that criterion at a given time point, information was set to zero. Values of *H* at *q* = 0 were used as an index of rate coding in cells with *H*_*max*_ > *H*_*shuffled*_+2SD. The average value of *H* at *q* = 0 and the average value of *H*_*max*_ across the entire sample were calculated at each time point, as well as the proportion of cells with *H*_*max*_ > *H*_*shuffled*_+2SD. To determine if differences existed in the cumulative proportional frequency distribution of the neurons utilizing temporal coding (either rate envelope or spike timing) in lean and DIO rats, a Kolmogorov-Smirnov test was used. An α = 0.05 was considered significant.

### Histology

To confirm the location of an electrode, animals were deeply anesthetized (Fatal Plus, Vortech Pharmaceuticals, Dearborn, MI, United States), and direct current (1 mA; 10–15 s) was passed through the electrode corresponding to the channel from which a taste-responsive neuron was recorded. Animals were then perfused transcardially with isotonic saline followed by a 10% paraformaldehyde solution. Brains were removed and placed in 10% formalin for a minimum of 1 week. Before sectioning, brains were rinsed with a phosphate buffer saline (PBS) three times, and then left in a 20% sucrose in PBS for a minimum of 24 h. Brains were then frozen and sectioned at a thickness of 35 μm through the medulla on a cryostat (Leica CM1850, Wetzlar, Germany). Tissue was thaw-mounted on slides (Colorfrost Plus, Thermo Fisher Scientific, Pittsburg, PA, United States), stained with cresyl violet, and the lesion/recording site was identified.

## Results

**FIGURE 1:**
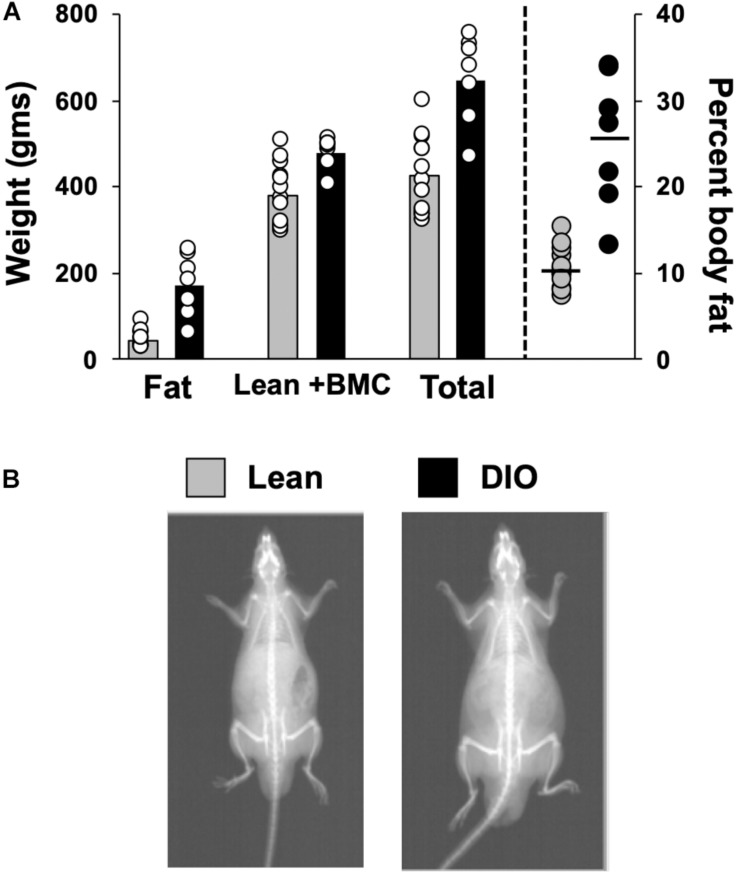
Body composition. **(A)** Scattergram of body composition data from lean and DIO rats. Left *Y*-axis: Animals’ fat mass in grams, lean plus bone mineral mass (BMC) content in grams, and total weight in grams. Right *Y*-axis: body fat percentage. Bars (left) and horizontal lines (right axis) indicate mean values and circles indicate values for individual rats. **(B)** DXA scan images of a lean (left) and obese (right) rat.

Body Composition data from 14 lean rats and 7 DIO rats revealed that rats fed a 45% high fat diet (HED) had a significantly different body composition compared to animals on a standard lab diet, *F*(1,54) = 62.0, *p* < 0.0001 (Figure 1). Rats on a HED had a higher total weight [*t*(54) = 6.81, *p* < 0.0001, 648.9 ± 38.9 g vs. 425.1 ± 24.8 g] and significantly more body fat than lean animals, [*t*(54) = 3.81, *p* < 0.0001, 170.9 ± 27.5 g of fat vs. 45.7 ± 5.3 g fat for DIO rats and lean rats, respectively]. As such, rats on a HED had a significantly higher body fat percentage, [*t*(54) = 6.5, *p* < 0.0001, 25.3 ± 2.9% vs. 10.4 ± 0.6%, for DIO and lean animals, respectively] compared to animals on a standard chow diet. There were no differences in lean body mass and bone mineral content, *t*(54) = 3.07, *p* = 0.06; 478.0 ± 13.6 g vs. 379.3.8 ± 19.9 g.

Electrophysiological taste responses were recorded from 74 cells in 14 lean animals and 49 cells in 7 diet-induced obese (DIO) animals. Recorded alongside these taste responsive cells were an additional 441 non-taste-responsive units in lean animals (total *N* = 515) and 179 non-taste-responsive units in DIO animals (total *N* = 228). The mean number of taste-responsive cells recorded per animal was 5.7 ± 2.2 in lean animals and 6.1 ± 1.1 in DIO animals, the median was 3 in lean animals, 5.5 in DIO animals. The range of recorded taste-responsive cells for lean animals was 1–30. The animal that provided 30 cells had 4 channels with taste-responsive activity for two consecutive days. The range of recorded taste-responsive cells for DIO animals was 3–10. For lean rats, data from 33 taste-responsive and 149 non-taste-responsive cells (total of 182 cells from 18 rats) were part of a dataset analyzed and published previously in Denman et al. (2019). In that study, analyses were focused on lick-related behavior, not taste-responsiveness, and data were culled from several electrophysiological studies with similar stimulus delivery protocols. The present data were thus part of a much larger dataset. Here, the focus of all analyses was solely on taste-responsive neurons.

**FIGURE 2:**
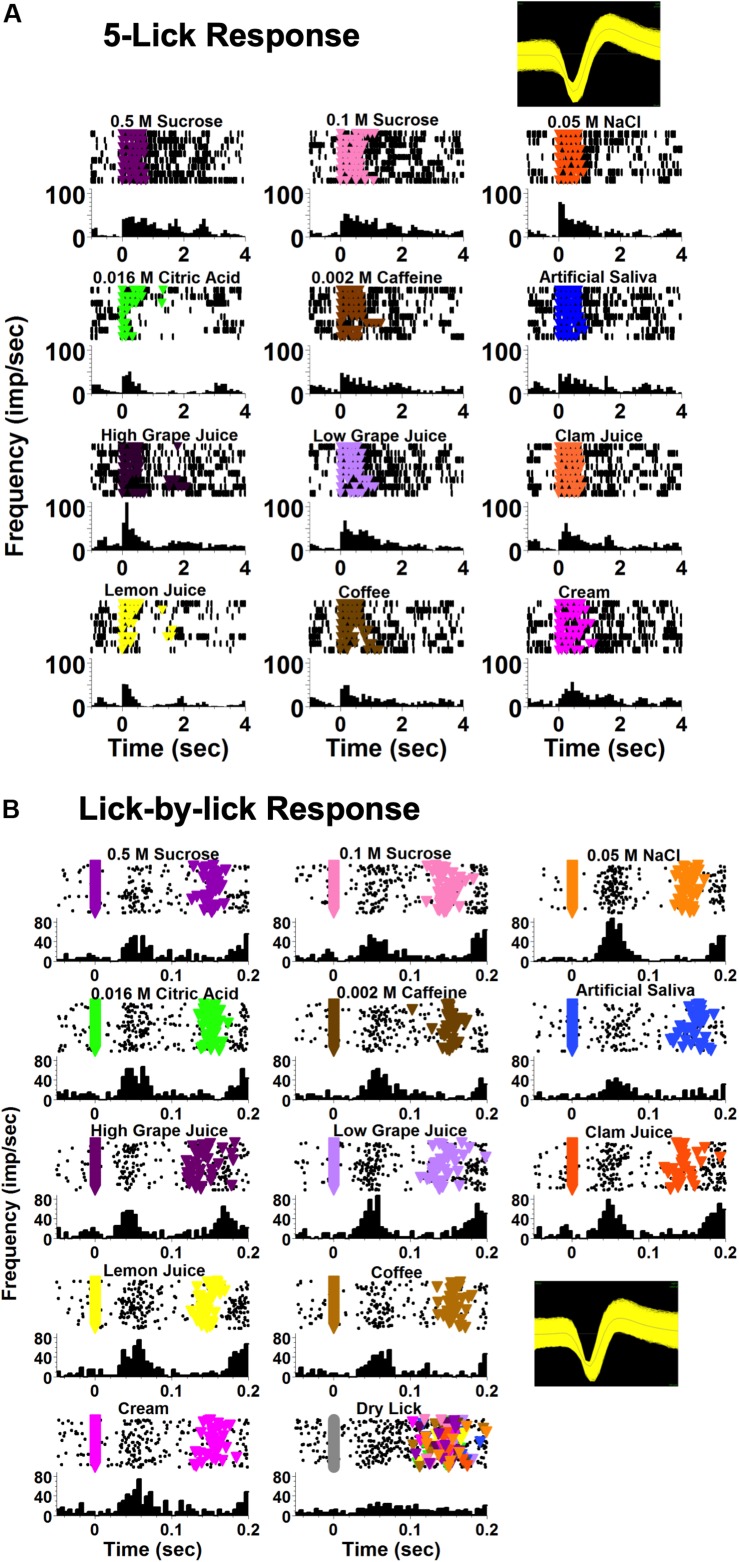
Examples of NTS taste responses on different time scales, recorded from two different cells. Both cells were recorded from a lean rat. **(A)** An example of a taste-responsive NTS neuron with a 5-lick response to taste. The first lick of the 5-lick stimulus trial is at zero; time bins are 100 ms. **(B)** An example of a taste-responsive NTS neuron with a lick-by-lick response. Each individual stimulus lick is positioned at zero; time bins are 5 ms. For both **(A,B)** each panel shows rasters (top) and peristimulus time histograms (PSTHs; bottom). Colored triangles in the rasters indicate a lick reinforced with a taste stimulus.

Taste-responsive cells in the NTS responded to taste stimuli at two different time scales. Some responses extended over, and sometimes past the 5-lick stimulus presentation; these were called “5-Lick responses.” Other responses occurred at a short latency (<100 ms) after each taste stimulus lick and were dissipated before the next lick; these were called “Lick-by-lick” responses. Figure 2 shows an example of these two types of taste response in two different NTS cells from lean rats.

**FIGURE 3:**
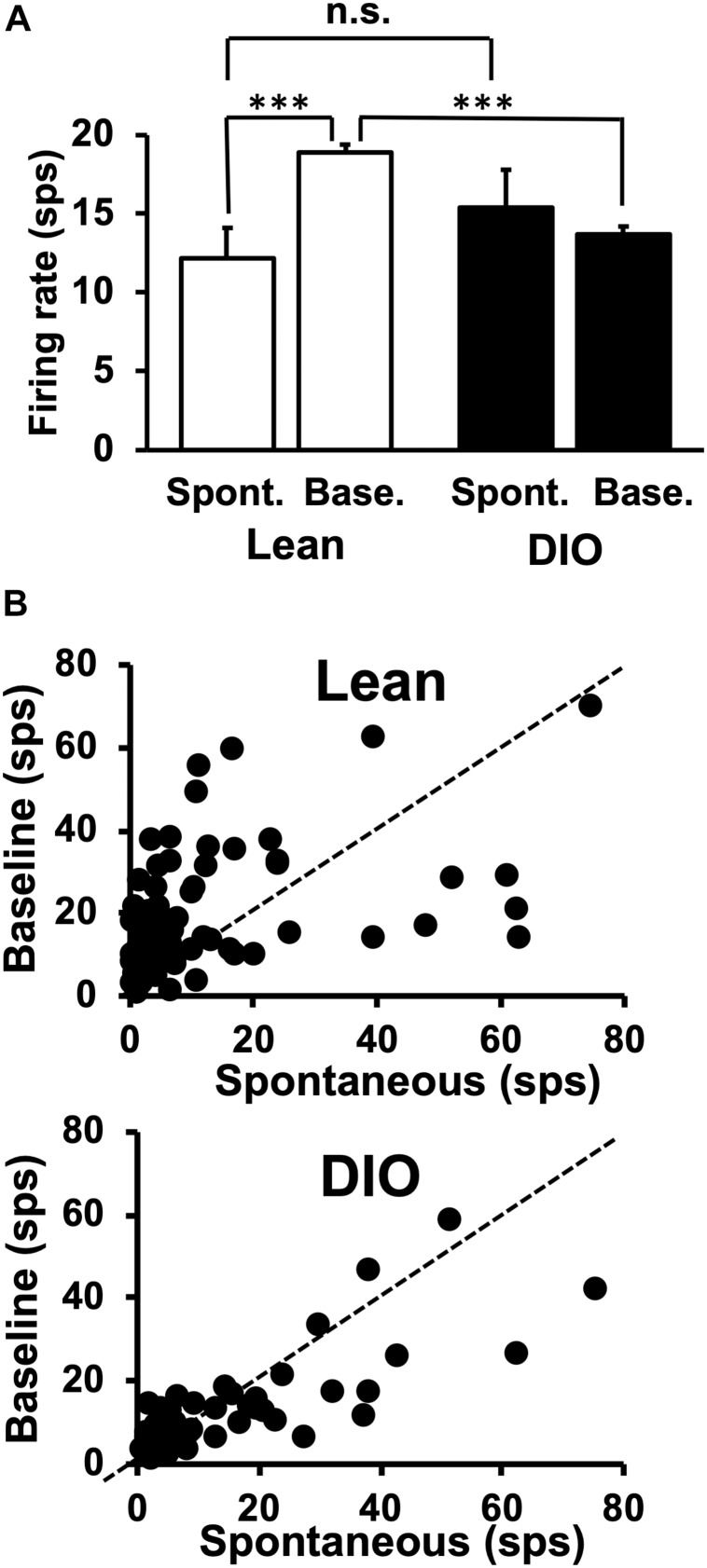
Spontaneous and baseline firing rates in NTS cells. **(A)** Mean firing rates are in spikes/sec ± SEM. Spontaneous firing rates are from 10 s of activity during which the animal did not lick. Baseline firing rates were calculated as the firing rate during the 1 s just prior to the first taste stimulus lick, during which period the rat was often emitting dry (unreinforced) licks. NTS cells in lean rats increase their firing rate when licking (spontaneous firing rate > baseline firing rate), but NTS cells in DIO rats do not. There was no significant difference between spontaneous rates in Lean vs. DIO rats (*p* = 0.29, indicated as n.s.) ^∗∗∗^*p* < 0.001. **(B)** Sacatterplots of spontaneous firing rate plotted against baseline firing rate for each cell in lean (top) and DIO (bottom) rats.

The average spontaneous firing rate was taken from multiple 14 s windows when the animal was not licking. The first 3 s of this window acted as a buffer to exclude any neural activity evoked by a previously presented taste stimulus; the 10 s period following the initial 3 s was used to calculate the spontaneous firing rate. The exclusion of the final 1 s in the spontaneous firing rate window ensured that there was at least 1 s between the time during which spontaneous activity was measured and any potential approach to the lick spout. Spontaneous firing rates are shown in Figure 3A and include data exclusively from taste-responsive cells. There was no significant difference in spontaneous firing rate between cells in lean animals [12.18 ± 1.93 spikes/sec (sps)] and neurons in DIO rats [15.4 ± 2.4 sps; *t*(121) = 1.05, *p* = 0.29].

Taste-responsive cells in lean animals showed larger baseline firing rates compared with those during spontaneous firing periods, but this was not true for cells in DIO rats. Unlike spontaneous firing rates calculated when the animal was not licking, baseline firing rates were calculated from a 1 s window before the first lick of a 5-lick taste stimulus trial. The baseline firing rates of taste responsive cells in DIO rats (13.7 ± 0.5 sps) were significantly lower than those in their lean counterparts [18.9 ± 0.5 sps; *t*(121) = 7.054, *p* < 0.0001] (see Figure 3B).

**FIGURE 4:**
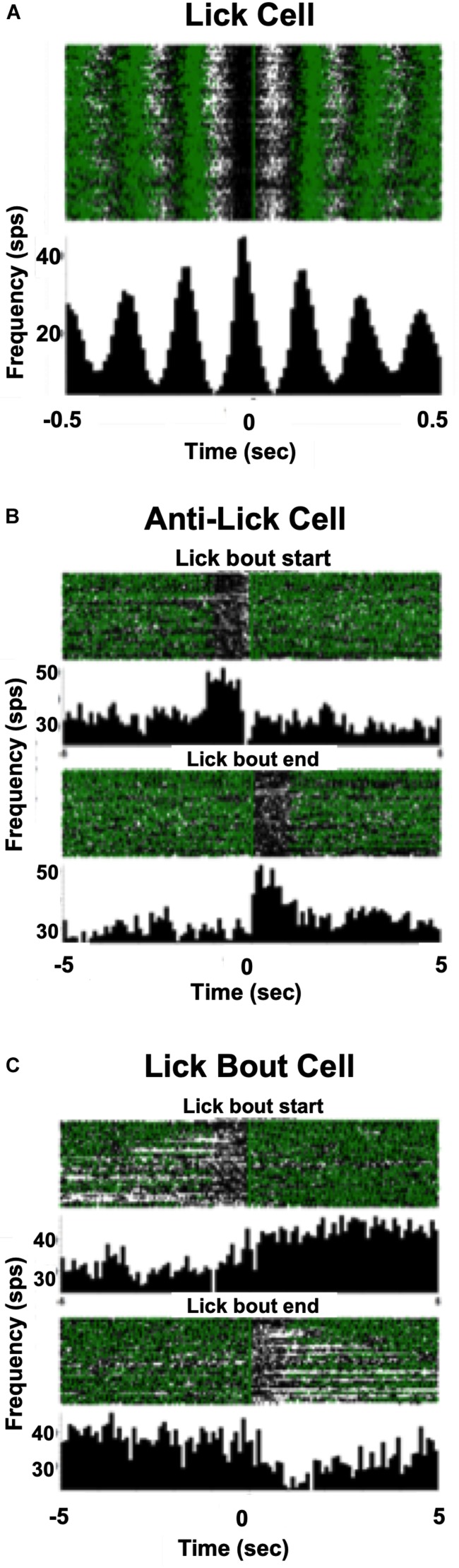
Examples of firing patterns of NTS cells. **(A)** Raster and PSTH of a neuron’s firing profile during licking. Each lick is positioned at *t* = 0; time bin = 10 ms. This cell increases its firing rate just before the occurrence of each lick. **(B)** Rasters and PSTHs of an anti-lick cell. The zero point is positioned just before the beginning of a lick bout (defined as at least 1 s of continuous licking), upper, or at the end of a lick bout, lower. Time bin = 100 ms. This cell shows a lower firing rate during a lick bout and a higher firing rate when the rat is not licking. **(C)** Rasters and PSTHs of a lick bout cell. The zero point is positioned just before the beginning of a lick bout, upper, or at the end of a lick bout, lower. This cell shows a higher firing rate during a lick bout and a lower firing rate when the rat is not licking. In **(A–C)** each green triangle indicates the occurrence of a lick.

**FIGURE 5:**
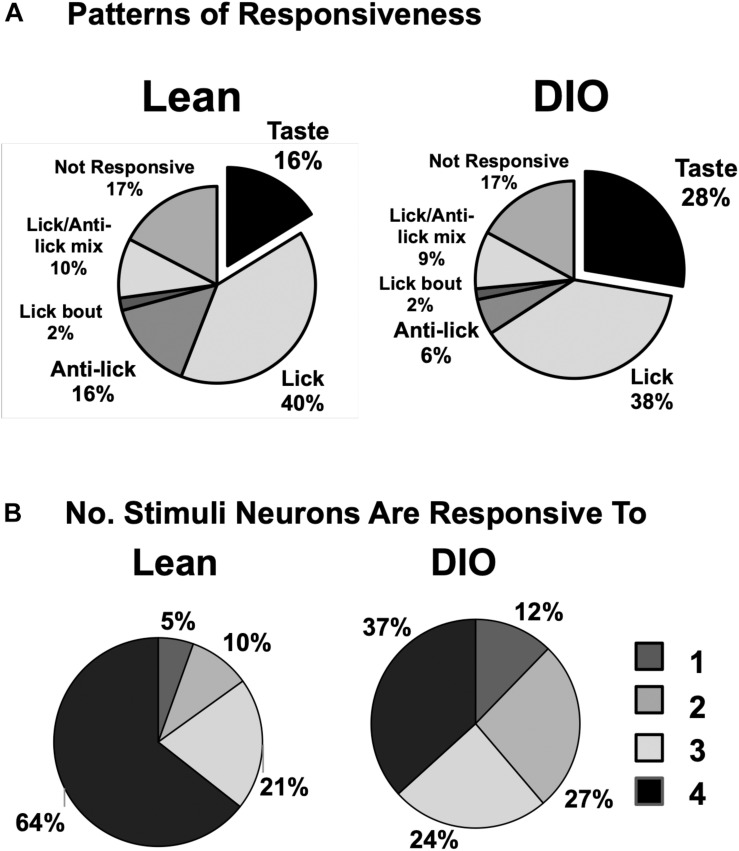
Response and tuning characteristics of NTS cells. **(A)** Proportion of NTS cells with various patterns of firing with respect to taste stimulus presentation and licking behavior. There is a larger proportion of taste-responsive cells in DIO rats compared to lean rats and a smaller proportion of anti-lick cells. **(B)** Tuning characteristics of taste-responsive NTS cells in lean and DIO rats. Shown is the proportion of cells that respond to one, two, three or four traditional tastants (0.1 M sucrose, 0.05 M NaCl, 0.016 M citric acid, and 0.002 M caffeine). Cells with 5-lick or lick-by-lick responses are included. NTS cells in DIO rats are generally more narrowly tuned than NTS cells in lean rats.

In addition to cells that increased their firing rate in response to one or more taste stimuli, there were several other firing patterns that we observed in both lean and DIO animals. “Lick cells” (*n* = 174 for lean; *n* = 65 for DIO) were defined as cells that fired in phase with the lick cycle; “anti-lick cells” (*n* = 65 for lean; *n* = 12 for DIO) were cells that showed a reduced firing rate during the lick-bout usually accompanied by a phasic increase in firing rate before and after a lick bout; “lick bout” cells (*n* = 9 for lean; *n* = 3 for DIO) were cells that increased their firing rate during a lick bout, but were not taste-responsive and did not fire in phase with licks; “lick / anti-lick mix cells” were cells that showed “anti-lick” properties, and also fired in phase with the lick cycle. Figure 4 shows examples of these patterns of firing. “Non-responsive cells” did not exhibit any changes in firing patterns either related to licking or taste stimulus presentation. Some taste-responsive cells also showed lick-related activity but were nevertheless classified here as solely taste-responsive. The distribution of the proportion of cells with various relationships to taste/licking as described above, shown in Figure 5A, was significantly different between lean and DIO animals [χ^2^ (5) = 14.33, *p* = 0.0136]. A greater proportion of cells in DIO animals were taste-responsive compared to the proportion of taste-responsive cells in lean rats (28% vs. 16%, respectively).

Figure 5B shows the distribution of taste-responsive cells that responded to one, two, three or four of the traditional taste stimuli (0.1 M sucrose, 0.05 M NaCl, 0.016 M citric acid, 0.002 M caffeine); both 5-lick and lick-by-lick responses were counted. Cells in both groups were generally broadly tuned, with only 5% of cells in lean rats and 12% of cells in DIO rats responding to only a single tastant. However, cells in DIO rats were significantly more narrowly tuned across taste qualities than cells in lean rats [χ^2^ (3) = 11.18, *p* = 0.0108].

**FIGURE 6:**
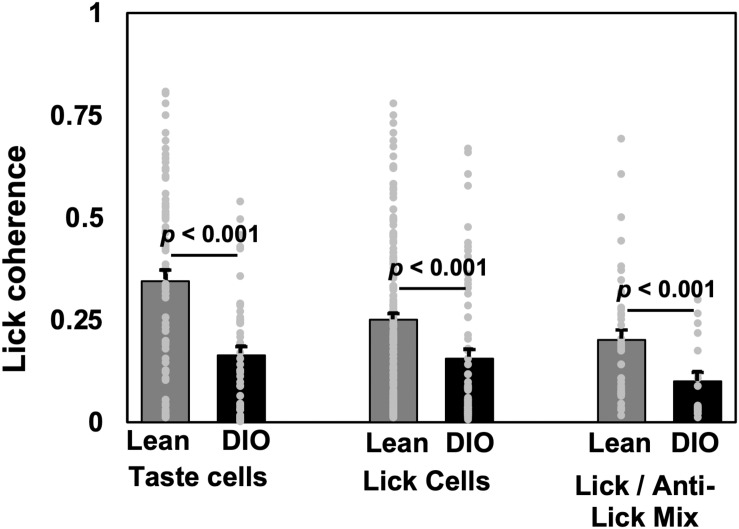
Lick coherence in taste-responsive, lick, and lick/anti-lick mix cells. Mean and individual values from each cell. Peak values of lick coherence within the 4–9 Hz range are shown. Cell numbers ae as follows: for lean rats, *n* = 74 taste cells, *n* = 172 lick-related cells, *n* = 43 anti-lick mix cells; for DIO rats, *n* = 49 taste cells, *n* = 66 lick-related cells, and *n* = 19 anti-lick mix cells. For all cell categories, NTS cells in DIO rats show significantly less lick coherence than NTS cells in lean rats.

Analyses of lick coherence showed that firing patterns in NTS cells in DIO rats were more loosely coupled to behavior than firing patterns in lean rats. Specifically, lick coherence values for neurons in lean animals were significantly different from those in DIO animals for taste cells (median = 0.345, *n* = 74 in lean, median = 0.163, *n* = 49 in DIO, *U* = 972, *p* < 0.0001), lick-related cells (median = 0.251, *n* = 172 in lean, median = 0.0596, *n* = 65 in DIO, *U* = 155, *p* < 0.0001), and lick/anti-lick mix cells (median = 0.201, *n* = 43 in lean, median = 0.100, *n* = 19 in DIO, *U* = 234, *p* = 0.0071). Figure 6 shows the average lick coherence plotted with the distribution of lick coherence values for taste, lick, and lick/anti-lick mixed cells.

**FIGURE 7:**
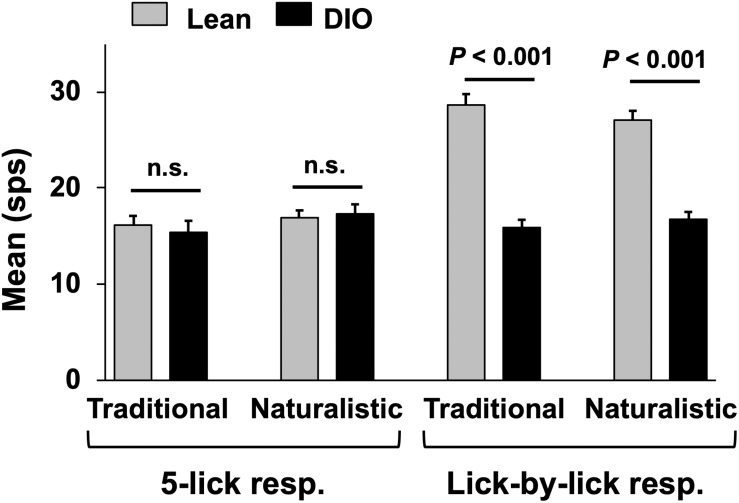
Mean (+SEM) response magnitude (in spikes per second; sps) for 5-lick (left) and lick-by-lick responses in NTS cells recorded from lean and DIO rats. Lick-by-lick responses in lean rats are significantly larger than those in DIO rats for both traditional and naturalistic tastants (Student’s *t* test, *p*s < 0.001).

**FIGURE 8:**
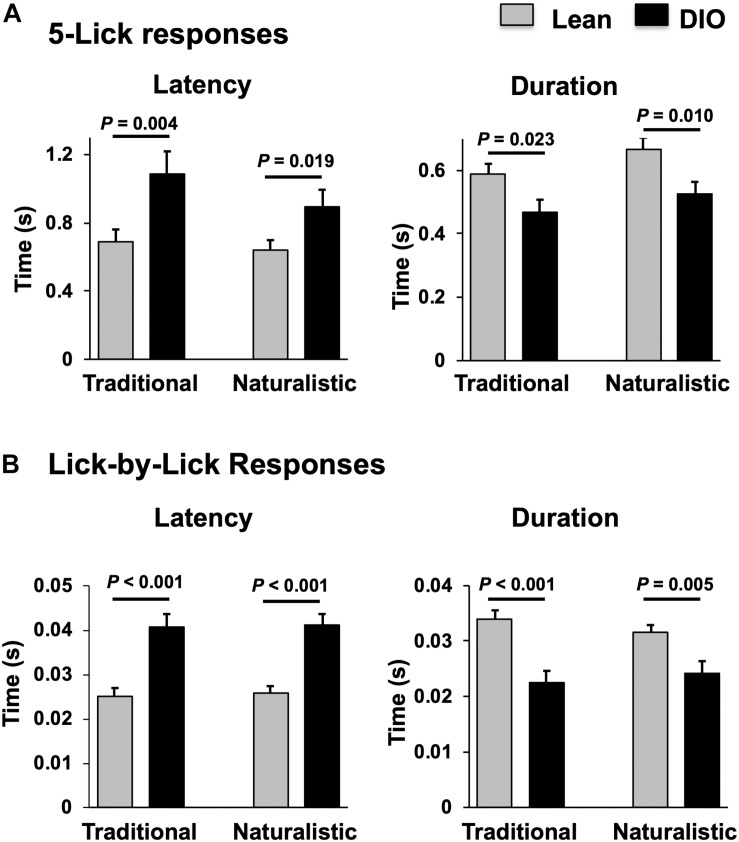
Comparison of latency and duration of NTS taste responses in lean and DIO rats. **(A)** Mean (+SEM) values of response latency (left) and duration (right) of 5-lick responses in lean and DIO rats. **(B)** Mean (+SEM) values of response latency (left) and duration (right) of lick-by-lick responses in lean and DIO rats. Values across lean and DIO groups were comparedusing a Student’s *t* test; *p* values are shown for each comparison. Both 5-lick and lick-by-lick NTS taste responses in DIO rats showed significantly longer latencies and shorter durations than those in lean rats.

Taste responses in NTS cells in the DIO rats are smaller in magnitude, shorter in duration and longer in latency than taste responses in NTS cells in lean rats. Table 1 shows mean ± SEM firing rates for all tastants. Although there was no effect of diet on the 5-lick response magnitude [*F*(1,412) = 0.066, p > 0.05], lick-by-lick taste responses were significantly smaller DIO vs. lean animals [*F*(1,913) = 100.4, *p* < 0.0001]. In addition, both 5-lick [*F*(1,513) = 13.38, *p* = 0.0003] and lick-by-lick [*F*(1,1006) = 15.08, *p* < 0.0001] taste responses in DIO rats were significantly shorter in duration than those in lean rats. Latencies of both 5- lick [*F*(1,510) = 20.13, *p* < 0.0001] and lick-by lick [*F*(1,1006) = 52.29, *p* < 0.0001] responses were longer in cells from DIO rats compared with those in lean rats. In all, there were no stimulus-specific significant differences that were detected. To illustrate these differences, taste responses were averaged separately across all traditional and naturalistic tastants and compared with a Student’s *t* test. Although there was no effect of diet on the 5-lick response magnitude [*t*(156) = 0.473, *p* = 0.637 for traditional tastants; *t*(247) = 0.324, *p* = 0.746 for naturalistic tastants], lick-by-lick taste responses were significantly smaller DIO vs. lean animals for both traditional [*t*(378) = 7.330, *p* < 0.001) and naturalistic tastants [*t*(473) = 6.56, *p* < 0.001; Figure 7]. In addition, latencies of both 5- lick [*t*(201) = 2.87, *p* = 0.004 for traditional tastants; *t*(294) = 2.36, *p* = 0.019 for naturalistic tastants] and lick-by lick responses [*t*(416) = 4.81, *p* < 0.001 for traditional tastants; *t*(504) = 5.35, *p* < 0.001 for naturalistic tastants] were longer in cells from DIO rats compared with those in lean rats (Figure 8). Similarly, both 5-lick [*t*(201), = 2.29, *p* = 0.023 for traditional tastants; *t*(294) = 2.59, *p* = 0.010 for naturalistic tastants] and lick-by-lick [*t*(416) = 4.05, *p* < 0.001 for traditional tastants; *t*(504) = 2.80, *p* = 0.005 for naturalistic tastants] taste responses in DIO rats were significantly shorter in duration than those in lean rats (Figure 8).

**TABLE 1:** Response magnitude (in sps).

	**Lean**						**DIO**					
		
	**Excitatory**			**Inhibitory**			**Excitatory**			**Inhibitory**		
				
**5-L**	**MEAN**	**SEM**	***N***	**MEAN**	**SEM**	***N***	**MEAN**	**SEM**	***N***	**MEAN**	**SEM**	***N***
Hi Su	13.9	2.0	21	−15.2	1.8	6	18.6	4.0	7	−10.5	2.3	3
Lo Su	14.2	1.8	24	−15.0	3.6	6	15.5	2.4	11	−10.2	1.8	3
N	16.0	1.9	19	−11.8	1.5	4	13.7	3.0	11	−6.7	0.3	3
CA	20.0	2.2	29	−16.9	2.5	8	15.8	2.3	17	−13.1	3.2	5
Caf	15.1	2.4	13	−12.9	3.3	5	10.8	2.4	7	−13.7	4.6	4
Hi GJ	15.7	2.1	27	−12.2	1.9	11	26.0	2.9	13	−10.1	3.4	3
Lo GJ	14.4	1.7	26	−9.3	7.0	5	14.7	2.1	15	−20.6	1	
Clm J	13.4	1.4	26	−18.2	3.1	5	13.5	4.0	8	−17.5	7.7	2
Lm J	20.4	2.0	28	−15.7	4.5	8	19.0	2.5	21	−8.0	1.8	2
Coff	18.5	1.8	26	−11.5	1.8	5	16.6	1.6	20	−11.1	3.2	4
Crm	18.2	2.1	21	−16.0	1.2	2	14.0	1.5	19	−9.9	3.8	3
AS	12.8	1.8	11	−19.7	4.8	3	14.0	2.0	8	−7.8	3.1	2

	**Lean**						**DIO**					
		
	**Excitatory**			**Inhibitory**			**Excitatory**			**Inhibitory**		
				
**LXL**	**MEAN**	**SEM**	***N***	**MEAN**	**SEM**	***N***	**MEAN**	**SEM**	***N***	**MEAN**	**SEM**	***N***
Hi S	29.4	2.6	54	−28.4	2.1	5	15.8	1.4	26	−14.5	1.8	5
Lo S	26.3	2.6	49	−19.6	2.7	6	15.0	1.6	25	−17.2	3.7	4
NaCl	31.0	2.6	50	−13.7	2.2	3	16.1	2.1	23	−19.7		1
CA	26.5	2.3	52	−19.5	5.0	5	15.5	2.3	25	−16.6	2.2	5
Caf	28.4	2.6	54	−37.3		1	17.3	1.6	23	−21.1	1.9	3
Hi GJ	28.4	2.6	52	−34.3	0.3	2	17.0	2.3	22	−21.9		1
Lo GJ	29.2	2.5	55	−22.4	4.5	3	19.0	1.9	22	−16.8	1.8	3
Clm J	30.0	2.5	56	−22.6	4.5	5	18.2	2.1	24	−10.6	4.2	4
Lm J	24.9	2.0	60	−29.0	4.9	3	14.6	1.7	25	−18.7	2.7	4
Coff	26.8	2.6	57	−25.8	9.5	2	16.1	2.3	20	−18.9	4.0	2
Crm	23.6	2.0	55	−22.9	7.8	2	16.0	1.2	27	−18.0	4.9	3
AS	25.9	2.5	57	−21.8	2.1	6	15.2	1.5	23	−6.4	1.2	12

*Lean *n* = 74; DIO *n* = 49. 5-L, 5-lick; LXL, lick-by-lick; Hi Su, 0.5 M sucrose, Lo Su, 0.1 M sucrose; N, 0.05M NaCl; CA, 0.01 M citric acid, Caf, 0.002 M; Hi GJ, 100% grape juice; Lo GJ, 25% grape juice; Clm J, clam juice; Lm J, lemon juice; Coff, coffee; Crm, 25% heavy cream; AS, artificial saliva.*

**FIGURE 9:**
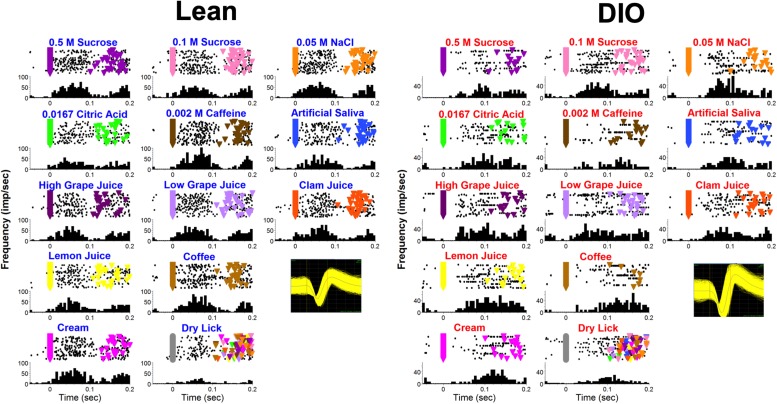
Examples of lick-by-lick taste responses in one unit from a lean rat (left) and another from a DIO rat (right). Top of each panel shows a raster; bottom of each panel shows a PSTH. Colored triangles indicate reinforced licks. Inset shows waveforms.

Figure 9 shows examples of lick-by-lick taste responses recorded from a lean (left) and DIO (right) rat. The responses in the neuron recorded from the DIO rat occur at a longer latency, are smaller in magnitude and shorter in duration than the responses in the neuron recorded from a lean rat, illustrating the main effects across the sample.

### Licking Behavior

Table 2 shows the results of analyses of licking behavior in DIO (A) and Lean (B) rats. Because the rats were licking taste stimuli for nearly the entire experimental session, the focus of these analyses were on licking patterns during taste stimulus trials. Since there were more taste-responsive cells recorded from lean compared with DIO rats, there were obviously more taste trials. In addition, there were proportionately more low sucrose trials for both DIO and lean rats. This was intentionally programmed into the session to maintain the motivation to lick, a tack that was especially important for DIO rats since they were often sluggish. In general, DIO rats licked at a slower pace than lean rats. This was evidenced by the significantly longer interlick intervals (by about 10 ms overall) in DIO rats (Wilcoxon signed rank test, *p* = 0.006) and a significantly longer time to complete all 5 licks of the taste stimulus (Wilcoxon signed rank test, *p* = 0.006), despite the fact that the intra-trial pause lengths were not significantly different across groups (Wilcoxon signed rank test, *p* = 0.178). DIO rats also accumulated significantly fewer trials per session than lean rats (Wilcoxon signed rank test, *p* < 0.001), again reflecting their slower lick rate.

**TABLE 2:** Analyses of licking behavior.

**Stimulus**	**Trials**	**ILI (s)**	**No. pauses**	**Pause length (s)**	**5 licks (s)**
**A. DIO rats**					
Hi Su	191 (6)	0.160	8	2.295	0.670
Lo Su	262 (9)	0.160	10	1.425	0.670
N	190 (6)	0.160	13	1.573	0.662
CA	203 (6.5)	0.170	113	2.530	1.245
Caf	192 (6)	0.170	9	1.400	0.708
Hi GJ	194 (6)	0.160	18	1.796	0.740
Lo GJ	194 (6)	0.168	17	1.452	0.770
Clm J	189 (6)	0.160	7	1.351	0.667
Lm J	202 (6)	0.170	158	2.378	1.693
Coff	191 (6)	0.170	66	2.070	0.901
Crm	186 (6)	0.170	37	1.664	0.715
AS	193 (6)	0.170	9	1.431	0.708
**B. Lean rats**					
Hi Su	347 (7)	0.153	2	1.165	0.627
Lo Su	442 (9)	0.151	2	1.090	0.620
N	353 (7)	0.152	0	N/A	0.625
CA	353 (8)	0.170	267	2.010	1.505
Caf	345 (7)	0.159	9	1.220	0.650
Hi GJ	340 (7)	0.144	27	1.586	0.647
Lo GJ	349 (7)	0.150	5	1.230	0.660
Clm J	352 (7)	0.150	1	1.900	0.618
Lm J	352 (7)	0.170	275	2.240	1.431
Coff	342 (7)	0.160	32	1.645	0.754
Crm	335 (7)	0.160	3	1.448	0.660
AS	346 (7)	0.160	4	1.728	0.652

*Abbreviations for taste stimuli are as in Table 1. Trials, total number of trials for each stimulus, number in parentheses is the median number of trials per session across animals; ILI, interlick interval; No. of pauses, the number of pauses within a 5-lick taste stimulus trial; Pause length, the median time (s) of pauses that occurred within a 5-lick taste stimulus trial; 5 licks, the time (s) to complete all five licks of a stimulus trial. All values are medians.*

### Analyses of Temporal Coding

Metric space analyses were applied to taste-evoked spike trains for the first 200, 500, 1000, 1500, and 2000 ms beginning with the first taste stimulus lick (see section “Materials and Methods” for details). For each cell at each response interval the analyses indicate the maximum amount of information about taste quality that is conveyed by the temporal characteristics of the spike train, termed *H*_*max*_, divided by the total number of cells. Only values of *H*_*max*_ that exceeded *H*_*shuffle*_ + 2SD were included. *H*_*count*_ indicates the amount of information about taste quality conveyed by the number of spikes that occurred during the respective response interval, irrespective of their timing.

**FIGURE 10:**
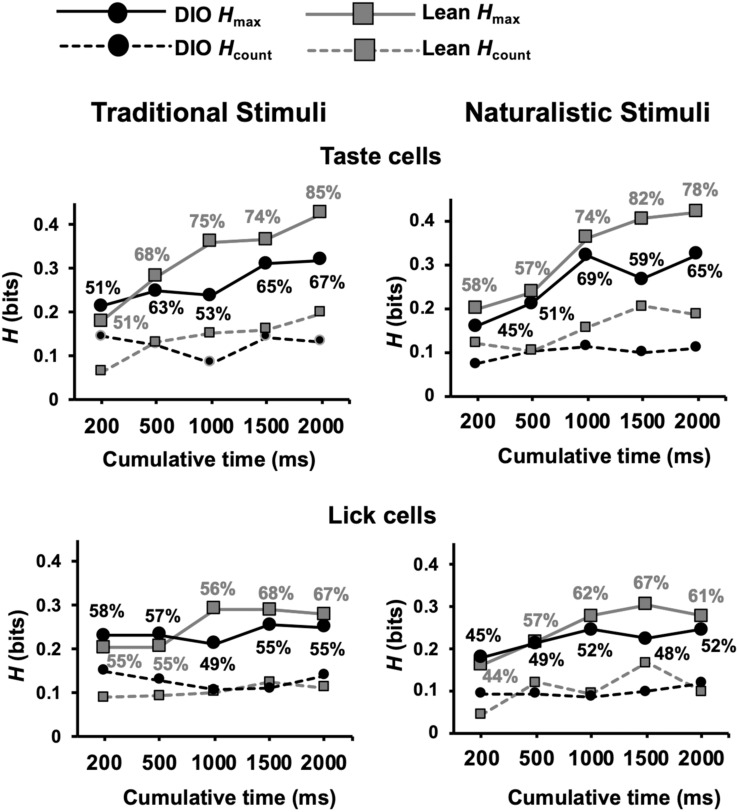
Results of metric space analysis of taste responses in DIO and lean rats. Results for taste-responsive cells are shown in top graphs for traditional (left) and naturalistic (right) stimuli. Similar results from lick-related cells are shown on bottom. Traditional taste stimuli included in the analyses were 0.1 M sucrose, 0.05 M NaCl, 0.01 M citric acid, and 0.002 M caffeine; naturalistic taste stimuli included were 25% grape juice, clam juice, lemon juice, and coffee. Percentages near each data point indicate the proportion of responses that conveyed a significant amount of information about taste quality (*H*_*max*_ > *H*_*shuffled*_+2SDs). *H*_*count*_ indicates the amount of information about taste quality conveyed by spike count alone. Perfect discrimination = 2 bits.

Results of metric space analyses show that the temporal characteristics of taste-evoked spike trains in DIO rats convey significantly less information about taste quality than taste-evoked spike trains in lean rats (see Figure 10). This was true for both traditional tastants and, in the longer response intervals, for the naturalistic tastants. In addition, a greater proportion of neurons in lean animals used temporal coding to convey taste-quality information compared to neurons in DIO rats for both traditional and naturalistic taste stimuli (Kolmogorov-Smirnov, *p* = 0.002). At 2 s after the first stimulus lick; this difference was especially pronounced for traditional stimuli, 85% of neurons in lean rats used temporal coding to convey taste quality information compared to only 67% of neurons in DIO rats. For both types of taste stimuli, the information derived from spike count was similar in DIO, and lean rats and was less than that when the temporal characteristics of the responses were considered.

Metric space analyses revealed a significant difference between lean and DIO animals in the proportion of lick-related cells that conveyed information about traditional taste stimuli (Kolmogorov-Smirnov, *p* = 0.008) (Figure 10). There was no difference between lean and DIO rats in the proportion of lick-related cells that exhibited temporal coding for naturalistic stimuli (Kolmogorov-Smirnov, *p* = 0.14). Thus, lick-related cells also convey information about taste quality, albeit less that that conveyed by taste-responsive cells.

**FIGURE 11:**
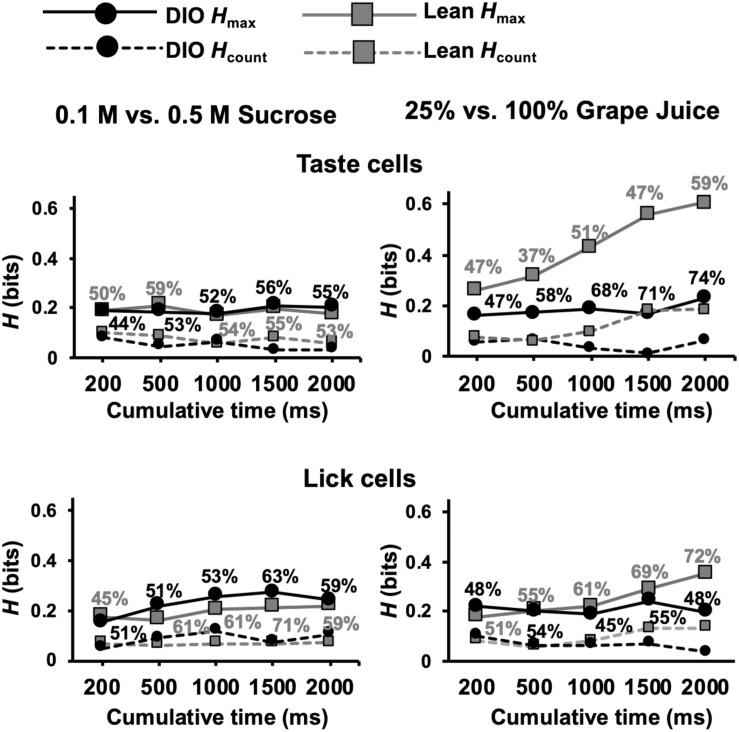
Results of metric space analysis of taste responses to high and low concentrations of sweet stimuli. Traditional taste stimuli included in the analyses were 0.1 M and 0.5 M sucrose; naturalistic stimuli included in the analyses included 25 and 100% grape juice. Percentages near each data point indicate the proportion of responses that conveyed a significant amount of information about sweet stimulus intensity (*H*_*max*_ > *H*_*shuffled*_+2SDs). *H*_*count*_ indicates the amount of information about taste quality conveyed by spike count alone. Perfect discrimination = 1 bit.

An additional set of analyses focused on the ability of cells in DIO and lean rats to distinguish between different concentrations of sweet tastants, specifically 0.5 M vs. 0.1 M sucrose and 25% vs. 100% grape juice. The rationale motivating these analyses came from previous electrophysiological (Kovacs and Hajnal, 2008) and behavioral (Hajnal et al., 2010) data indicating a rightward shift in the sucrose concentration-response function in the brainstem as well as reduced intake of sucrose in obese compared to lean rats. We therefore hypothesized that taste-responsive cells in the NTS of DIO rats would convey less information about sucrose intensity than lean rats. Contrary to this conjecture, Figure 11 shows that, for different concentrations of sucrose, cells in both DIO and lean rats conveyed similar amounts of information (Mann Whitney *U* test, *p*s > 0.05); however, for different concentrations of grape juice, cells in DIO rats conveyed significantly less information than cells in lean rats, especially when longer response intervals (1.5 and 2 s) were considered (Mann Whitney *U* test, *U* = 1210 and 1273, respectively; *p*s ≤ 0.0135). Additionally, lick-related cells in lean animals were able to convey more information about the naturalistic sweet stimuli compared to those in DIO animals at 2 s (*U* = 3123; *p* = 0.0004), but not before (*p*s > 0.1). Importantly, these results were not a reflection in the lick pattern, as there was no significant difference in the information conveyed by lick pattern between lean and DIO rats (Supplementary Figure 1).

### Histology

**FIGURE 12:**
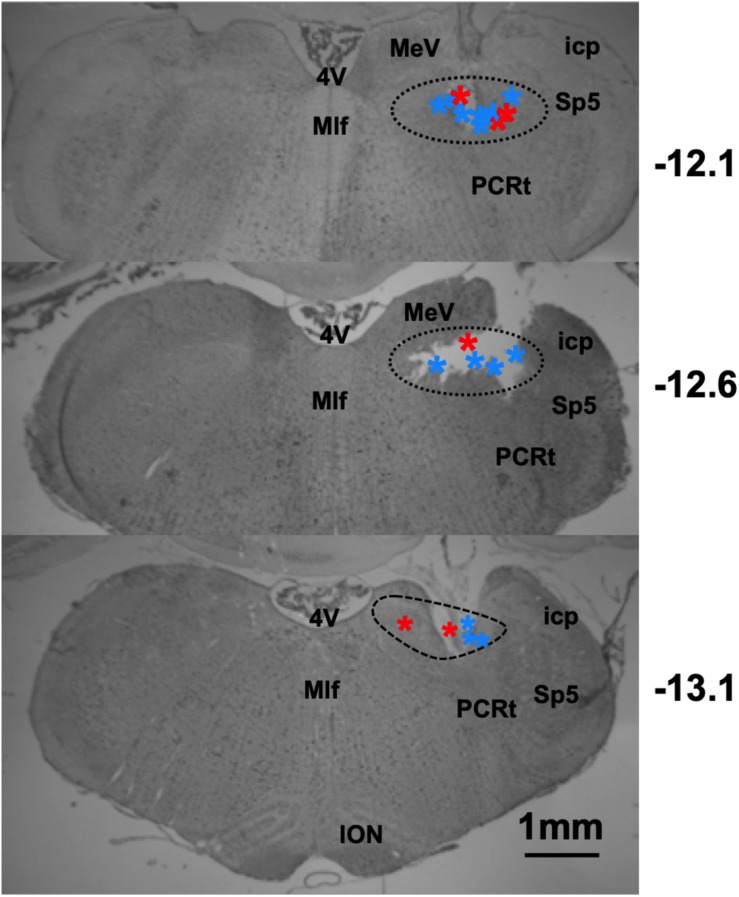
Results of histological analyses reconstructing recording locations in lean and DIO rats. Photomicrographs of coronal sections through the rat brainstem. Dashed lines indicate the area of the NTS. Stars indicate lesion site, red = DIO rats, and blue = lean rats. Sp5, spinal trigeminal nucleus; PCRt, parvocellular reticular nucleus; 4V, 4th ventricle; ION, inferior olivary nucleus; MeV, medial vestibular nucleus; Mlf, medial longitudinal fasiculus; icp, inferior cerebellar peduncle.

The locations of taste-responsive cells were determined by passing 1 mA of current for 10 s through the wire that corresponded to the last channel where a taste-responsive cell was recorded. Lesion locations, as shown in Figure 12, spanned from 12.1–13.1 mm posterior, and between 1 and 2.2 mm lateral to Bregma.

## Discussion

Electrophysiological recordings from single cells in the NTS of awake DIO and lean rats show that exposure to a HED can alter the ways in which taste is encoded. Specifically, responses to taste stimuli in DIO rats were smaller in magnitude, shorter in duration, and occur at longer latencies compared with those in lean rats. Not surprisingly then, taste-responsive cells in the NTS of DIO rats are more narrowly tuned and convey significantly less information about taste quality than taste-responsive cells in lean rats. An additional 620 cells were recorded alongside taste-responsive cells in the NTS of DIO and lean rats, the great majority of which showed firing patterns that closely tracked licking behavior. These lick-related cells also conveyed information about taste quality, though less than that conveyed by taste-responsive cells. NTS cells in DIO rats also showed less lick coherence than those in lean rats suggesting a disconnect of spike activity and behavior in the NTS of DIO rats. Changes in the relative proportion of taste-responsive and other cells as a result of a HED may be a compensatory mechanism for blunted taste responses. In all, the present data reveal that a HED can have profound effects on how cells in the brainstem represent taste stimuli. These effects in turn may ultimately impact food choice and body weight.

The attenuation of taste responses in the NTS of DIO vs. lean rats may be the natural response to a weaker input from the periphery. Previous reports have detailed reduced taste receptor cell expression (Zhang et al., 2011, 2013; Kaufman et al., 2018) and reduced activation of taste receptor cells (Maliphol et al., 2013) with obesity. These results may contribute to the relatively narrow tuning of NTS cells in DIO vs. lean rats. This is likely the consequence of smaller response magnitudes overall; that is, weaker input may not adequately drive taste responsivity in cells from DIO rats, resulting in fewer significant taste responses, and narrower tuning. Relatedly, in humans with obesity, taste-evoked potentials are shorter and weaker (Hardikar et al., 2018) compared to those in lean control subjects. In that paper, the authors showed that taste perception did not differ across obese and lean groups, but the reward value of palatable sucrose was suppressed in obese subjects. Collectively, these data suggest that the shorter duration of taste responses in obese subjects, both rodents (present data), and humans (Hardikar et al., 2018) might underlie the lower hedonic responses to palatable taste stimuli in overweight subjects that have been reported previously (Bartoshuk et al., 2006). In addition, lower taste sensitivity has been reported to correlate with a higher basal metabolic index (Overberg et al., 2012; Vignini et al., 2019).

Results also showed that there were proportionately more taste-responsive cells in the NTS of DIO vs. lean animals. This suggests the possibility that more NTS cells may be recruited to respond to taste to compensate for weakened taste responses. Obviously, the conversion of non-taste-responsive into taste-responsive cells would require that there were many NTS cells that had the requisite connectivity available but normally ineffective. Such potentiality of NTS cells in the short term has been shown following adaptation (Di Lorenzo and Lemon, 2000) and following brief “pre-pulses” of tastants (Di Lorenzo et al., 2003) where taste responses appeared where none were previously. Changes in metabolic state, such as obesity, may unmask taste responses on a longer time scale.

Taste responses to all tastants, regardless of palatability, were attenuated in DIO vs. lean rats. That is, the effects of a HED in the NTS were not restricted to any subset of tastants but were instead a general characteristic of taste-responsive cells in NTS. This observation apparently conflicts with data showing that genetically obese rats show selective changes in taste preference for sweet, but not salty, sour, or bitter tastants (Hajnal et al., 2010). This ostensible disconnect of physiology and behavior may reflect anatomical differences in the parts of the gustatory neural circuit that process taste or food identification vs. preference. In particular, taste preference may be processed, and evident, upstream in the central gustatory pathway. For example, electrophysiological responses to sweet tastes in the parabrachial nucleus of the pons (the second synapse in the central gustatory pathway and the main target of NTS output) are selectively affected in genetically obese rats, consistent with behavioral preference profiles (Kovacs and Hajnal, 2008).

### Temporal Coding

As taste responses develop over time, the cumulative amount of information about stimulus identity conveyed by spike trains progressively increases in both lean and DIO animals. However, for traditional taste stimuli, this information was smaller for DIO compared with that in lean rats at all time points, and significantly smaller at 1 and 2 s. This result may be another reflection of a weaker drive from the periphery (Kaufman et al., 2018). The shorter duration of responses in DIO rats implies that taste responses in these rats may trial off without conveying the amount of information that taste responses in their lean counterparts convey with longer lasting responses. Consistent with that idea is the observation that the information about taste quality conveyed in DIO and lean rats was similar for the earlier parts of the responses but diverged at longer time intervals, where presumably information continued to accrue in lean rats but not in DIO subjects. Additionally, the proportion of neurons that use temporal coding to convey taste quality information was larger in lean rats than obese rats for both traditional and naturalistic stimuli. These results are consistent with the general finding that the neural representation of taste quality in the NTS of DIO rats is weaker, and thus less competent to encode taste-related information, than that in lean rats.

It can be argued that, unlike traditional taste stimuli, naturalistic tastants contain volatiles that provide olfactory input which stimulates, and/or modulates NTS taste responses (Escanilla et al., 2015). The added olfactory information offered by naturalistic tastants may facilitate stimulus discrimination in both DIO and lean rats (Escanilla et al., 2015). This was apparent when animals were presented with a sweet stimulus at two different concentrations. There was no difference between NTS cells in DIO vs. lean rats in the amount of information discriminating two concentrations of sucrose. However, NTS cells in DIO animals were impaired in their ability to convey information about two different concentrations of grape juice, despite the fact that the sugar concentrations in the grape juice were matched to those of sucrose. It is possible that NTS cells in lean animals were able to take advantage of information from other modalities (e.g., olfaction) stimulated by grape juice but not sucrose, to discriminate different stimulus intensities, but NTS cells from DIO rats could not. This notion would be consistent with reports showing deficits in olfactory-driven behaviors in DIO rats (Lacroix et al., 2015).

### Firing Patterns in Taste Cells Reflect Behavior

In the gustatory cortex, the state of the neural network when information about a taste stimulus arrives is adjusted to optimize encoding of that stimulus (Yoshida and Katz, 2011). Specifically, behavioral tasks that require active sensory acquisition modulate both pre-stimulus activity and taste selectivity of single neurons (Yoshida and Katz, 2011). Consistent with these results are data showing that cues that predict taste stimulation can lead to firing rate changes prior to taste presentation as well as a decrease in the taste response latency (Samuelsen et al., 2012; Gardner and Fontanini, 2014). These data show that the animal’s cognitive state, and therefore the state of the neural network encoding taste stimuli can influence the ability of neurons to convey taste-related information. In the present dataset, network activity both pre-sensory acquisition and during the acquisition phase was indexed by spontaneous and baseline firing rates, respectively. Spontaneous firing rates did not differ significantly in NTS cells in DIO vs. lean rats; however, in lean rats, there was an increase in firing rate when the rats transitioned from spontaneous firing to sensory acquisition, where the rat was actively licking. A similar shift in firing rate did not occur in NTS cells in DIO rats, suggesting that the neural network in the NTS of DIO rats did not adjust to sensory acquisition as did the NTS in their lean counterparts. Moreover, when a lean animal initiates licking, firing rates in anti-lick cells are suppressed, offering taste-responsive cells a high signal-to-noise environment in which to convey information about taste stimuli. However, in DIO rats, there were proportionately fewer anti-lick cells than in lean rats, so taste responses in DIO rats, already relatively weak, and occurred in a relatively noisy background compared with those in lean rats.

Lick-related activity is present throughout the rodent gustatory neuraxis (Stapleton et al., 2006; Gutierrez et al., 2010, 2011; Weiss et al., 2014; Fonseca et al., 2018), including the NTS (Roussin et al., 2012; Denman et al., 2019), and no doubt reflecting and influencing network-wide activity. The synchronous neural activity afforded by lick entrainment over several taste-related neural structures may enhance cross-structure communication (Fries, 2015; Bonnefond et al., 2017). Lick-related activity in the NTS of the awake rat has been found to be widespread and informative with respect to taste quality discrimination (Denman et al., 2019). Data reported here agree with these results. However, NTS cells in DIO rats showed less lick coherence than those in lean rats. Lick-related activity enhances the neural representation of taste and that without it, taste stimuli are less well discriminated, as seen in the NTS of DIO rats reported here. This relative disconnect of taste sensation and ingestive behavior buttress the dysfunctional, blunted taste signal that is relayed upstream in the brain.

## Conclusion

Present data show that the neural processing of taste stimuli in the NTS of DIO rats is impaired. Taste responses in DIO rats are blunted and convey less information than those in lean rats. Shifts in the relative proportions of various patterns of ingestion-related firing may represent the system’s adaptive mechanism aimed at compensating for a compromised taste system. The changes noted here may provide some insight into the mechanism(s) underlying altered taste perception in humans with obesity (Bartoshuk et al., 2006; Pepino et al., 2010; Sartor et al., 2011; Noel et al., 2017). Although there is evidence that weight loss improves taste perceptions in humans with obesity (Scruggs et al., 1994; Miras and le Roux, 2010), it is unclear whether the changes found in the NTS of DIO animals can also be reversed. Further research will address this question.

## Data Availability

The datasets generated for this study are available on request to the corresponding author.

## Ethics Statement

The animal study was reviewed and approved by the Binghamton University Institutional Animal Care and Use Committee.

## Author Contributions

MW collected the data. MW and PD carried out the data analyses, prepared the figures, and wrote the first draft of the manuscript. KC, AH, and PD edited the manuscript.

## Conflict of Interest Statement

The authors declare that the research was conducted in the absence of any commercial or financial relationships that could be construed as a potential conflict of interest.
