# Supplementary figures and images for: Taste Responses in the Nucleus of the Solitary Tract of Awake Obese Rats Are Blunted Compared With Those in Lean Rats
Source: Front Integr Neurosci. 2019 Jul 30;13:35. doi: 10.3389/fnint.2019.00035 (PMC6683675; doi:10.3389/fnint.2019.00035)

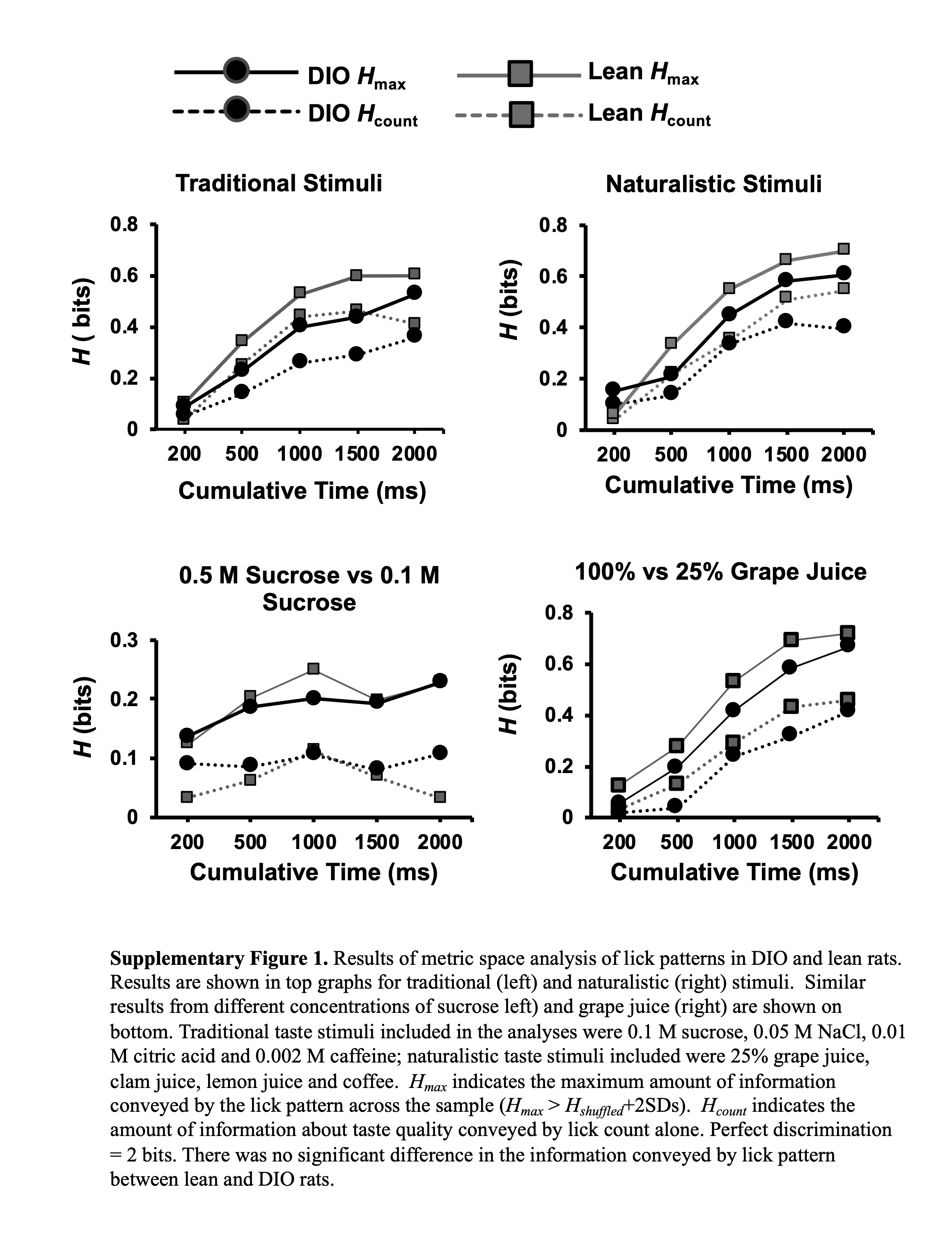

Supplement: Supplementary file 1 [file Image_1.TIFF]
